# A model combining clinical and genomic factors to predict response to PD-1/PD-L1 blockade in advanced urothelial carcinoma

**DOI:** 10.1038/s41416-019-0686-0

**Published:** 2019-12-20

**Authors:** Amin H. Nassar, Kent W. Mouw, Opeyemi Jegede, Atul B. Shinagare, Jaegil Kim, Chia-Jen Liu, Mark Pomerantz, Lauren C. Harshman, Eliezer M. Van Allen, Xiao X. Wei, Bradley McGregor, Atish D. Choudhury, Mark A. Preston, Fei Dong, Sabina Signoretti, Neal I. Lindeman, Joaquim Bellmunt, Toni K. Choueiri, Guru Sonpavde, David J. Kwiatkowski

**Affiliations:** 1 Department of Medical Oncology, Dana-Farber Cancer Institute, Boston, MA USA; 2 Department of Medicine, Brigham and Women’s Hospital, Boston, MA USA; 3 Department of Radiation Oncology, Dana-Farber Cancer Institute, Boston, MA USA; 4 Department of Biostatistics and Computational Biology, Dana-Farber Cancer Institute, Harvard Medical School, Boston, MA USA; 5 Department of Radiology, Brigham and Women’s Hospital, Boston, MA USA; 6 The Broad Institute of MIT and Harvard, Cambridge, MA USA; 7 Division of Urology, Brigham and Women’s Hospital, Boston, MA USA; 8 Department of Pathology, Brigham and Women’s Hospital, Boston, MA USA; 9 Department of Oncologic Pathology, Dana-Farber Cancer Institute, Boston, MA USA; 10 Department of Medical Oncology, IMIM-Hospital del Mar Medical Research Institute, Barcelona, Spain

**Keywords:** Predictive markers, Bladder cancer

## Abstract

**Background:**

In metastatic urothelial carcinoma (mUC), predictive biomarkers that correlate with response to immune checkpoint inhibitors (ICIs) are lacking. Here, we interrogated genomic and clinical features associated with response to ICIs in mUC.

**Methods:**

Sixty two mUC patients treated with ICI who had targeted tumour sequencing were studied. We examined associations between candidate biomarkers and clinical benefit (CB, any objective reduction in tumour size) versus no clinical benefit (NCB, no change or objective increase in tumour size). Both univariable and multivariable analyses for associations were conducted. A comparator cohort of 39 mUC patients treated with taxanes was analysed by using the same methodology.

**Results:**

Nine clinical and seven genomic factors correlated with clinical outcomes in univariable analysis in the ICI cohort. Among the 16 factors, neutrophil-to-lymphocyte ratio (NLR) ≥5 (OR = 0.12, 95% CI, 0.01–1.15), visceral metastasis (OR = 0.05, 95% CI, 0.01–0.43) and single-nucleotide variant (SNV) count < 10 (OR = 0.04, 95% CI, 0.006–0.27) were identified as independent predictors of NCB to ICI in multivariable analysis (c-statistic = 0.90). None of the 16 variables were associated with clinical benefit in the taxane cohort.

**Conclusions:**

This three-factor model includes genomic (SNV count >9) and clinical (NLR <5, lack of visceral metastasis) variables predictive for benefit to ICI but not taxane therapy for mUC. External validation of these hypothesis-generating results is warranted to enable use in routine clinical care.

## Background

Immune checkpoint inhibitors (ICIs) have transformed the therapeutic landscape of a growing list of human cancers,^1–3^ including metastatic urothelial carcinomas (mUC).^4–7^ However, only 20–30% of mUC patients respond to ICIs, and an even smaller proportion achieve durable responses lasting ≥2 years.^8^ Hence, mUC remains an incurable disease for the majority of patients due to inherent or acquired therapeutic resistance. The mechanisms underlying variation in ICI response among mUC patients are poorly understood, and there is an urgent clinical need to identify biomarkers that are predictive of ICI benefit and to elucidate the mechanisms of resistance in ICI nonresponders.

Currently, the only Food and Drug Administration-approved predictive biomarkers of ICI response are programmed death-ligand (PD-L1) expression for specific cancers and microsatellite instability-high (MSI-H)/mismatch repair deficiency (dMMR) for a tumour-agnostic indication. However, although PD-L1 expression has some value for prediction of response to ICIs, it is not consistent across different ICIs and lines of therapy.^5,9–11^ Mismatch repair deficiency is associated with clinical benefit in several tumour types,^9,12^ but is rare in mUC. Tumour mutational burden (TMB) and predicted neoantigen load have also been correlated with ICI response in several tumour types, including non-small-cell lung cancer and melanoma.^1,13–17^ However, the clinical applicability and predictive power of TMB is uncertain.^18^ In mUC, low TMB does not preclude response and high TMB is not sufficient to predict response.^19^

Recently, DNA damage repair (DDR) gene alterations were reported to be associated with response to ICIs in patients with mUC in a single-institution series.^19^ Larger prospective cohorts are required to validate this potential biomarker. In addition, peripheral blood markers have emerged as potential biomarkers of ICI response in multiple cancer types. Low absolute neutrophil count, low neutrophil-to-lymphocyte ratio (NLR) and low absolute monocyte count were associated with improved overall and progression-free survival in melanoma patients receiving ipilimumab (anti-CTLA-4).^20^ In addition, a recently reported six-factor prognostic model for overall survival in advanced UC patients treated with post-platinum atezolizumab consisted of two clinical factors (ECOG PS ≥1 vs. 0, presence of liver metastasis) and four blood-based biomarkers (anaemia, thrombocytosis, NLR ≥ 5 and elevated LDH).^21^ In this study, we sought to develop an integrated model combining genomic, clinical and routine laboratory factors to predict response to anti-PD-1/PD-L1 inhibitors in mUC regardless of the setting (i.e. first-line or post platinum).

## Methods

### Study design and patient cohort

We identified patients with histologically confirmed diagnosis of mUC treated with an anti-PD-1/PD-L1 agent at Dana-Farber Cancer Institute (DFCI) between June 2013 and December 2017 who also underwent tumour DNA sequencing analysis (see below) (Fig. S1). Mutational findings in these patients were recently reported but were not analysed for response to immune checkpoint therapy.^22^ A radiologist (A.S.), blinded to genomic and clinical data, performed tumour measurements using Response Evaluation Criteria in Solid Tumors version 1.1 (RECIST v1.1). Both computerised axial tomography and positron emission tomography scans were used to assess measurable lesions (total of five lesions and maximum of two per organ) that met RECIST v1.1 criterion. With prior reports suggesting a need for enhanced clinical endpoints to assess clinical benefit in patients treated with ICIs,^23^ patients were classified as having clinical benefit (CB) if they had any objective reduction in tumour burden, and having no clinical benefit (NCB) if they had any progressive disease or no reduction in tumour burden. This study was approved by the Institutional Review Board of DFCI.

A parallel cohort of 39 mUC patients who received taxane-based chemotherapy, but did not receive ICIs, was also analysed by similar methods.

### Data collection

Clinical variables that were assessed included gender, race, age, smoking status, prior systemic chemotherapy regimens, prior radiotherapy, primary tumour site and site of lesion subjected to targeted sequencing. Baseline Eastern Cooperative Oncology Group (ECOG) performance status (PS), liver/visceral metastasis, haemoglobin (Hb), NLR and platelet (PLT) count were captured at the time of ICI initiation (Table 1, S1.0).

**Table 1:** Clinical characteristics of the mUC patients who received ICI, and were assessed for response.

	Number (%), [*n* = 62]
*Clinical/demographic variables*	
Age, years	
Mean, range	65.6, 41.0–84.0
Gender	
Male	45 (73)
Female	17 (27)
ECOG PS	
0	26 (44)
1	25 (42)
≥2	8 (14)
Missing	3
Site	
Bladder	46 (74)
Upper tract	16 (26)
Radical cystectomy/nephrouretrectomy	
Yes	43 (69)
No	19 (31)
Previous lines of systemic therapy	
0	15 (24)
1	36 (58)
≥2	11 (18)
Visceral metastases (bone, lungs, liver, etc.)	
Yes	45 (73)
No	17 (27)
Neutrophil/lymphocyte ratio	
Mean, range	5.0, 0.5–18.3
Haemoglobin (g/dl)	
Mean, range	12.0, 8.0–16.5
Platelet count	
Mean, range	240, 60–588
*Genomic factors*	
SNV count	
Mean, range	9.7, 1.0–32.0
CNV count	
0	29 (47)
1	19 (31)
≥2	14 (23)
C>T_CpG mutation signature count	
Mean, range	2.6, 0.0–9.5
ERCC2 mutation signature count	
Mean, range	2.2, 0.0–7.8
APOBEC mutation signature count	
Mean, range	4.0, 0.0–19.5
*CDKN2B*	
Homozygous deletion	14 (23)
No	48 (77)
DNA damage repair (DDR) gene alteration (inclusive approach)	
Yes	34 (55)
No	28 (45)

### Tissue collection and DNA extraction

Core biopsy and/or surgical resection specimens were reviewed by a BWH genitourinary pathologist (SS) to confirm the diagnosis, histological subtype, tumour grade and stage. Tumour regions consisting of at least 20% tumour cells were macrodissected from unstained slides, and DNA was isolated using the QIAamp DNA FFPE Tissue Kit (Qiagen) according to the manufacturer’s instructions. DNA quantification was performed by Nanodrop and Pico-Green assays.

### Tumour-targeted gene sequencing

Targeted gene sequencing was performed using an institutional analytic platform, Oncopanel, that is certified for clinical use and patients reporting under the Clinical Laboratory Improvement Amendments (CLIA) act. Genomic DNA from each tumour sample was subjected to targeted exon capture and sequencing using one of three versions of the Oncopanel assay (V1–V3) in the Department of Pathology at Brigham and Women’s Hospital (BWH). The Oncopanel gene panel includes capture probes for 275–447 cancer-associated genes, as well as intronic portions of 60 genes for rearrangement detection.^24^ We focused our mutational and copy-number variation (CNV) analysis on 237 genes that were common to all versions of Oncopanel (Table S1.1).

The mean depth of read coverage for the targeted genes was 294× (Table S1.0). The single-nucleotide variant (SNV) count was defined as the number of exonic non-synonymous mutations per sample, including indels, nonsense mutations, splice site mutations and non-synonymous missense variants. In other reports, this is often referred to as tumour mutation burden (TMB).

### Variant assessment

We did not have sequencing information for germline DNA as part of this study. We excluded tumour sequence sequencing variants that were observed at a frequency >0.1% in the Exome Aggregation Consortium (ExAC) database,^25^ as they were considered likely germline variants. All loss-of-function variants were considered deleterious, including nonsense mutations, frameshift indels or splice site alterations affecting consensus nucleotides. The functional impact of missense mutations was determined using SIFT^26^ and Polyphen-2.^27^ Missense mutations classified as “damaging” in SIFT and/or “probably damaging” in Polyphen-2 were deemed deleterious (Table S1.3). Oncopanel covers 30 DNA damage repair (DDR) genes previously described in the literature (Table S1.4).^28^ Special consideration was given to missense variant assessment for DDR genes since prior studies have reported an association with benefit in the setting of chemotherapy or ICIs.^19,29–31^ For DDR genes, we used two different strategies to define significant mutations. The first, a more inclusive method, included all loss-of-function variants as well as missense variants judged significant by either Polyphen-2^27^ or SIFT,^26^ as described above (Table S1.5). The second, a more restrictive definition, included all loss-of-function alterations, and missense variants that met any of the following criteria: seen at least five times in the Catalogue of Somatic Mutations in Cancer (COSMIC) database,^32^ reported in OncoKB^33^ or reported in cancerhotspots.org^34^(Table S1.6). For *ERCC2*, all missense alterations within or near conserved helicase domains^35^ were included.

### Mutation signature analysis

SNVs in the 62 samples were classified into 96 base substitution types within the trinucleotide sequence context that includes the bases immediately 5′ and 3′ to each altered base. Mutation signature analysis was performed to resolve the SNVs for each sample into a set of characteristic patterns (signatures) to infer the contributions of each signature in each tumour.^36^ The SNVs for each sample were projected onto the four mutation signatures (APOBEC-a, APOBEC-b, ERCC2 and C>T transitions at CpG dinucleotides) known to occur commonly in bladder carcinoma.^37^ This yielded a count of the estimated number of mutations in each sample generated by each of the four mutational processes (Table S1.7).

### Copy-number variant (CNV) analysis

CNVs were identified using a custom R-based tool (VisCap-Cancer)^28^ that compares read depth at all genomic regions assayed among different samples. We focused on the most reliable CNVs in this analysis, homozygous deletions and amplifications, the latter defined as >6 copies.^38,39^ We calculated the CNV count as the total number of these two CNV events for each sample, considering only homozygous deletions in tumour-suppressor genes and amplifications in proto-oncogenes (Table S1.8).

### Statistical analysis

Statistical tests included the Chi-Square and Fisher’s exact tests for categorical variables and the Mann–Whitney *U* test (two-group comparisons) or the Kruskal–Wallis test (three-group comparisons) for continuous variables. Associations between nine clinical and seven genomic features and clinical outcomes were assessed using univariable and multivariable binary logistic and Cox regression model. The primary clinical outcome was to examine the associations with clinical benefit. Secondary outcomes included overall survival (OS), and progression-free survival (PFS). OS was calculated from the start date of ICI therapy to the date of death or the last follow-up. Patients alive were censored at the date of the last contact. Progression-free survival (PFS) was calculated from the start date of ICI therapy to the date of progression, death or the last follow-up. Patients alive and progression-free were censored at the date of the last disease assessment.

Multivariable binary logistic (for NCB vs. CB) and Cox (for PFS and OS) regression models were fitted to the data using the variables selected by the ALASSO method.^40^ Regression coefficients were estimated in univariable analysis, separately for clinical and genomic variables, using binary logistic (for NCB vs. CB) and Cox proportional hazards (for PFS and OS) regression models, for each variable selected by the ALASSO method. Using a two-sided *p* ≤ 0.05 criterion, variables from univariable analyses were selected for inclusion in the multivariable model with a stay criterion of ≤0.10; the same stay criterion was used for the combined model containing clinical and genomic variables. Model discrimination performance was assessed using the area under the ROC curve (AUC), referred to as c-statistic (or c-index) for PFS and OS. See supplementary material 1 for additional details on model building and statistical analysis.

## Results

### Patient and treatment characteristics

From June 2013 through December 2017, 102 mUC patients received PD-1/PD-L1 inhibitors at our institution and 67 of these patients had targeted next-generation tumour sequencing (Oncopanel) analysis performed on a primary or metastatic specimen. Of these, 62 patients were considered evaluable for response and were included in the analysis (Table 1, Fig. S1). The median patient age was 67 years (range, 41–84 years) and the majority of patients were male (73%). Fifteen patients (24%) were treated with ICI as first-line treatment for metastatic disease, while the remaining patients received chemotherapy prior to an ICI. Most patients received the anti-PD-L1 agent atezolizumab (61%) or the anti-PD-1 agent, pembrolizumab (31%; Table S1.0). The majority of samples that were analysed by sequencing were primary tumours (46/62, 74%), and were obtained prior to the patient receiving an ICI (57/62, 92%; Table S2). Twenty-four (39%) patients had CB in response to ICIs, while 38 (61%) did not. A separate cohort of 39 patients who received a taxane were identified (Table S2.0, Table S2.1). Seventeen (44%) patients received docetaxel-based regimens, while the remaining 56% were treated with paclitaxel-based regimens. Sixteen patients received sequential taxane and ICI therapy, and 11 of these 16 had discordant clinical benefit between ICIs and taxanes.

### SNV count/TMB is associated with clinical benefit to ICI

**Fig. 1:**
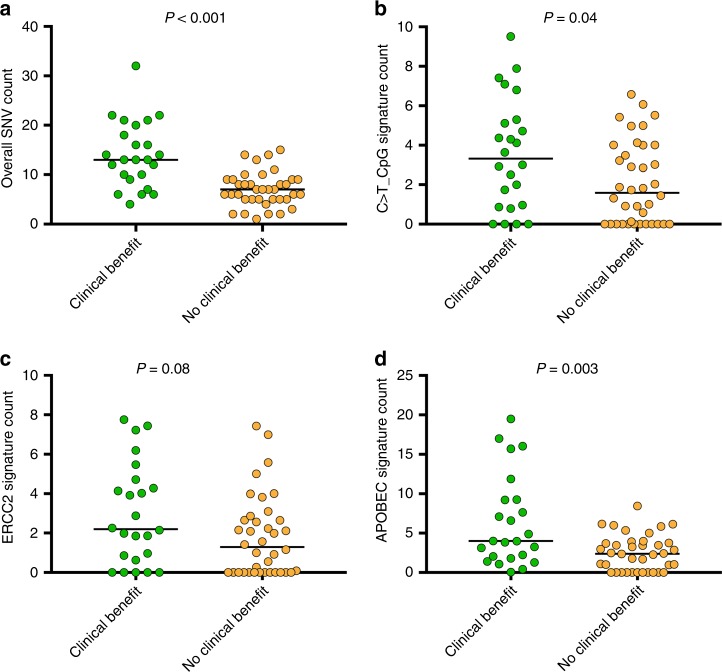
Association between SNV count and clinical benefit to ICI in mUC. Dot plots are shown for **a** overall SNV count, **b** C>T@CpG signature mutation count, **c** ERCC2 signature mutation count and **d** APOBEC signature mutation count. Each dot represents a patient.

The association between single-nucleotide variant (SNV) count, determined by Oncopanel analysis (see the “Methods” section), and ICI response was examined. A median of 8 SNVs were identified in the 62 tumours analysed (range 1–32 SNVs). Patients with CB had a significantly higher SNV count than patients with NCB (CB: median 13, range 4–32 vs. NCB: median 7, range, 1–15, *p* < 0.001, Fig. 1a). Higher SNV count was also associated with longer progression-free survival (median PFS 6.01 vs. 1.97 months for patients with ≥ median SNV count vs. those with < median SNV count, respectively, *p* = 0.002).

### APOBEC mutagenic signature is also associated with CB to ICI

The mutation signature, or pattern of specific mutations seen, in a tumour is a reflection of the different mutational processes active during its development. In the TCGA muscle-invasive bladder cancer cohort,^37^ two mutational signatures associated with apparent aberrant activity of the APOBEC cytosine deaminase accounted for 67% of all SNVs and were strongly associated with TMB.^37^ To investigate the relationship between mutational signature activity and ICI response, we projected all mutations in our cohort onto the three most common mutation signatures (fusing together two closely related APOBEC signatures) defined in the TCGA data. The number of estimated C>T_CpG mutations and estimated APOBEC-related mutations were both significantly higher in mUC patients with CB to ICI, whereas the number of estimated ERCC2 mutations showed a trend to be higher in mUC with CB to ICI (*p* = 0.041, 0.003 and 0.078, respectively, Mann–Whitney *U* test, Fig. 1b–d). However, none of the three mutational signatures were associated with PFS or OS (Table 2).

**Table 2:** Univariable analysis of clinical and molecular factors associated with CB vs. NCB, PFS and OS.

	Clinical benefit (odds ratio for CB vs. NCB)	PFS, hazard ratio	OS, hazard ratio
	Univariable (95% CI) [*p*-value]	Univariable (95% CI) [p-value]	Univariable (95% CI) [p-value]
Age	–	–	1.02 (0.98 1.06) [0.42]
Previous lines (≥1 vs. 0)	–	–	0.89 (0.33 2.44) [0.83]
Neut./lymph ratio *Continuous variable*	0.77 (0.60 0.99) [0.04]	–	1.12 (1.01 1.24) [0.03]
Haemoglobin *Continuous variable*	1.54 (1.10 2.17) [0.01]	0.77 (0.65 0.91) [0.002]	0.69 (0.54 0.88) [0.003]
Platelet count *Continuous variable*	0.997 (0.991 1.003) [0.32]	1.003 (1.00 1.01) [0.07]	1.003 (1.00 1.01) [0.16]
Tract (bladder vs. UTUC)	2.31 (0.65 8.24) [0.20]	0.62 (0.32 1.21) [0.16]	1.53 (0.52 4.53) [0.44]
Gender	0.82 (0.26 2.61) [0.73]	1.24 (0.63 2.44) [0.53]	1.13 (0.46 2.77) [0.80]
ECOG PS (≥1 vs. 0)	0.14 (0.05 0.46) [0.001]	3.00 (1.48 6.06) [0.002]	6.19 (1.93 19.86) [0.002]
Visceral/LN Mets.	0.03 (0.01 0.17) [<0.001]	6.91 (2.44 19.57) [<0.001]	14.49 (1.91 110.07) [0.010]
*Genomic variable*			
SNV count/TMB *Continuous* variable	1.36 (1.16 1.60) [<0.001]	0.88 (0.82 0.95) [ < 0.001]	0.94 (0.86 1.02) [0.14]
CNV count *Continuous variable*	0.40 (0.20 0.81) [0.01]	1.42 (1.11 1.81) [0.01]	1.12 (0.78 1.63) [0.54]
C>T CpG signature mutations *Continuous variable*	–	0.89 (0.78 1.02) [0.11]	0.96 (0.81 1.15) [0.69]
ERCC2 signature mutations *Continuous variable*	–	–	0.95 (0.77 1.16) [0.60]
APOBEC signature mutations *Continuous variable*	1.31 (1.09 1.58) [0.004]	–	0.91 (0.81 1.03) [0.15]
CDKN2B homozygous deletion	0.08 (0.01 0.69) [0.02]	3.84 (1.93 7.61) [<0.001]	4.12 (1.64 10.34) [0.003]
DDR deleterious alterations (Yes vs. No)	3.07 (0.98 9.59) [0.14]	–	0.61 (0.23, 1.62) [0.55]

–Variable with 0 coefficients from penalised regression

### CNV count and homozygous CDKN2B deletions are associated with NCB to ICI

Since aneuploidy is associated with reduced immune-mediated cytotoxic function,^41,42^ and CNV counts are associated with reduced survival in other cancer types,^43,44^ we examined the association between CNV count (see “Methods” section) and clinical outcomes in this cohort. The median CNV count was one (range 0–4). Higher CNV counts were seen in patients with NCB compared with patients with CB (*p* < 0.001, Mann–Whitney *U* test, Fig. S2A). Although there is a potential confounding effect of tumour purity on our measurement of CNV, we did not find an association between tumour purity and CB (Fig. S2D). Nonetheless, there was a significant association between CNV count and tumour purity (*p* = 0.012, Fig. S2B), but not SNV count (Fig. S2C), consistent with an effect of tumour purity on the sensitivity of detection of CNV events. Higher CNV count was also associated with poorer PFS (hazard ratio [HR], 1.42; 95% confidence interval [CI], 1.11–1.81; *p* = 0.01, Table 2), but not OS.

**Fig. 2:**
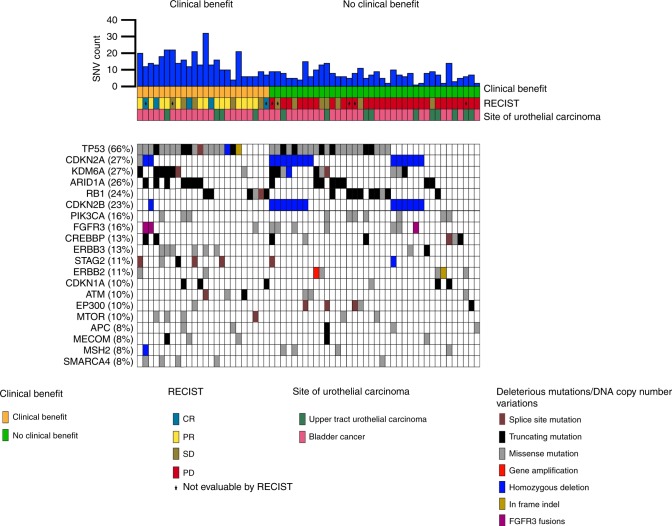
Co-mutation plot for UC patients treated with ICI. The 20 most frequently altered genes in this cohort are shown. CR, complete response; PR, partial response; SD, stable disease; PD, progressive disease.

Homozygous deletion of *CDKN2A* and *CDKN2B* was the most common CNV event in this cohort. *CDKN2A* and *CDKN2B* are located in close proximity (within 50 kb) on chromosome 9p, and their loss was concordant in the majority of samples (Fig. 2, Table S1.8). Homozygous deletions in *CDKN2A* and *CDKN2B* were each strongly associated with NCB in this cohort, with a slightly stronger effect for *CDKN2B* homozygous deletions (OR for NCB = 0.08; *p* = 0.02, Table 2). In addition, *CDKN2B* homozygous deletion was significantly associated with both worse PFS (HR = 3.84; *p* < 0.001) and OS (HR = 4.12; *p* = 0.003).

### Interaction between SNV and CNV counts and response to ICI

**Fig. 3:**
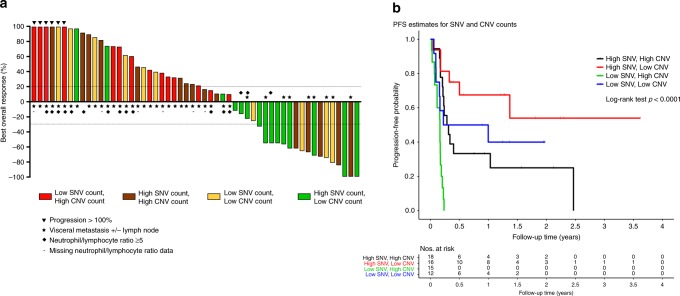
Response to ICI according to SNV and CNV count, and clinical features. **a** Waterfall plot of response to ICI therapy, indicating SNV count, CNV count, the presence of visceral metastasis and NLR. **b** Progression-free survival (PFS) curve of UC patients in response to ICI, based on median cut-offs for SNVs (8) and CNVs (0 vs. >0).

Next, we considered SNV and CNV counts in combination and determined their association with response. Patients were classified into four subsets based on SNV (high or low) and CNV (high or low) counts, using the median of each count to divide the groups (Fig. S3). In total, 13 of 17 (76%) patients with a combination of high SNV (≥8) and low CNV(0) had CB, while none of 15 with low SNV (<8) and high CNV ( > 0) counts had CB. Patients with high SNV and CNV, or low SNV and CNV had intermediate response rates, 6 of 18 (33%) and 5 of 12 (42%), respectively (*p* < 0.001, Chi-square test). Despite the small sample sizes, these combined SNV–CNV subsets demonstrated a highly significant difference in PFS (*p* < 0.0001, Fig. 3).

### DDR genes and response to ICI therapy

DDR gene alterations have been reported to be associated with response to ICIs in mUC.^19^ In our cohort, we used two approaches to define significant mutations in DDR genes, one more inclusive and the other more restrictive (see “Methods” section). Using the more inclusive criteria, DDR gene alterations were identified in 34 of 62 (55%) patients (Table S1.5). Tumours harbouring one or more DDR gene alterations had a significantly higher SNV count than tumours without DDR gene alterations (*p* = 0.013; Mann–Whitney *U* test; Fig. S4). Given this correlation with SNV count, we studied the association between DDR gene alterations and clinical benefit using both the inclusive and restrictive criteria. Using both methods, we observed a trend towards enrichment of DDR gene alterations in the CB group, although statistical significance was not attained (*p* = 0.192, *p* = 0.129, respectively, Fisher’s exact test).

When individual DDR pathways were considered separately, both homologous recombination (HR) and nucleotide excision repair (NER) pathways demonstrated enrichment for mutations (using the more inclusive approach) in the CB versus NCB groups (7/24, 29% vs. 1/38, 3%, *p* = 0.004; 5/24, 21% vs. 1/38, 3%, *p* = 0.029, respectively). Mutations in several individual genes were also enriched in the CB group: *ERBB3* (6/24, 25% vs. 2/38, 5.3%, *p* = 0.047), *MSH6* (4/24, 17% vs. 0/38, 0%, *p* = 0.019) and *BRCA1* (3/24, 5% vs. 0/38, 0%, *p* = 0.054). However, because of the large number of genes evaluated for this association, none of these were significant after correction for multiple testing.

### Association of clinical factors with ICI response

The presence of visceral metastases showed the strongest association with NCB (OR for NCB = 0.03, *p* < 0.001, Table 2) among all clinical variables considered. High NLR, low haemoglobin and low ECOG PS (≥1) were also all associated with NCB (OR = 0.77, *p* = 0.04; OR = 1.54, *p* = 0.01; OR = 0.14, *p* = 0.001, respectively; Table 2). NLR was associated with PFS, and haemoglobin, ECOG PS and visceral metastases were all strongly correlated with both PFS and OS (Table 2).

### Models combining clinical and genomic factors for clinical benefit

To develop a robust predictor of clinical benefit to ICI therapy, we combined the molecular, clinical and laboratory factors associated with benefit in a single analysis and performed a multivariable analysis using ALASSO (see “Methods” section) to define independent predictors of response. Lack of visceral metastases, NLR <5 and high SNV count (≥10) were all significantly associated with CB versus NCB, with a c-statistic (95% CI) of 0.90 (0.80, 0.99) (Table 3, Fig. S5). These three factors along with the CNV count for each patient and the extent of response to ICI according to RECIST 1.1 criteria are shown in Fig. 3a.

**Table 3:** Multivariable model for CB versus NCB, containing clinical and genomic factors.

Variable	Beta ± SE	OR (95% CI)	*p*-value	Points	c-statistic (95% CI)
Model intercept	1.15 ± 0.97	3.16 (0.47, 21.14)	0.237	1.0	0.90 (0.80, 0.99)
Visceral/LN Mets.	−2.93 ± 1.06	0.05 (0.01, 0.43)	0.006	−2.5	
Neut/lymph ratio (≥5 vs. <5)	−2.11 ± 1.14	0.12 (0.01, 1.15)	0.066	−2.0	
SNV count (≥10 vs. <10)	3.21 ± 0.97	24.79 (3.73, 164.70)	<0.001	3.0	

A linear predictor of clinical benefit was calculated using the three independent predictors of response (visceral metastasis, NLR and SNV count) including the intercept term (Table S4.1). In this predictor, the baseline was +1, visceral metastases scored as –2.5, NLR ≥5 as –2 and SNV count ≥10 as +3, giving a range of scores from –3.5 to 4 (Table 3, S4.1). A threshold of ≥ –1 was determined to be optimal for prediction of CB. Excluding 7 patients due to lack of NLR data at the time of ICI initiation, 22 of 24 patients with CB had a point score ≥ –1 (sensitivity = 92%, Table S4.2), while 27 of 31 patients with NCB had point scores < –1 (specificity = 87%, Table S4.2). Interestingly, no patient with visceral metastasis, NLR ≥5 and SNV count <10 (*n* = 12) had clinical benefit to ICI therapy (point score = –3.5, Table S4.1). In contrast, patients without visceral metastasis, NLR <5 and SNV ≥10 (*n* = 10) all derived clinical benefit to ICIs (point score = 4, Table S4.1).

### Predictive value of the ICI response model

To assess whether the association between these three factors and response to ICI therapy was prognostic or predictive, we evaluated all 16 variables found to be prognostic on univariable analysis in the ICI cohort in a comparator group of 39 patients from our institution treated with taxanes (Table S2.5). None of these 16 variables were significantly associated with response to taxanes (Table S2.5).

### Predictive model combining clinical and genomic factors for survival

Multivariable composite models for PFS and OS were also developed. Visceral metastasis, platelet count and both SNV and CNV counts were independent predictors of PFS (c-statistic (95% CI) = 0.77 (0.75, 0.79), Table S5.1). A prognostic index for PFS was calculated including these four components (see Supplemental Material 1 for details). Patients were divided into three risk groups based on their prognostic index score (low risk < –0.29; –0.29≤ intermediate risk <1.54; high risk ≥1.54), and PFS varied significantly among risk groups (Fig. S6A).

NLR, visceral metastasis and ECOG PS (≥1 vs. 0) were independent predictors of OS (c-statistic (95% CI) = 0.85 (0.83, 0.87), Table S5.2), all of which were categorical. Hence, a point system was used to calculate an OS predictor based on the coefficient of each risk factor (visceral metastasis, point score of 2; neutrophil-to-lymphocyte ratio, 1; ECOG PS, 1). Patients were then classified into three subgroups (0–2 points, 3 points and 4 points), which had very different observed OS (Fig. S6B).

## Discussion

In this study, we found that SNV count (tumour mutational burden, TMB) was significantly and strongly predictive of clinical benefit among mUC patients treated with ICI therapy with an odds ratio of 1.36 (1.16–1.60, 95% CI) and *p* < 0.001. These results are consistent with the known/suspected mechanism of action of anti-PD-L1 and anti-PD-1 therapies, which are thought to enhance productive neoantigen presentation to T cells, leading to T-cell attack and tumour shrinkage.^45^

We also showed an association of high NLR with lack of clinical benefit to ICIs. The mechanism of this association, which has been reported in other ICI studies,^46^ is debated. The ratio is elevated by higher levels of circulating neutrophils and/or lower levels of lymphocytes, and both of those events may contribute to a lack of response to ICI therapy. Neutrophilia may correlate with neutrophil abundance in cancers, which can contribute to a pro-tumour microenvironment by secreting VEGF, MMP-9^47^ and reactive oxygen species.^46,48^ PD-L1 expression on the surface of infiltrating neutrophils may also inhibit the activation of T cells.^49^ Reduced levels of circulating lymphocytes may correlate with reduced T-cell lymphocyte levels in tumours, which are required for an effective T-cell response to tumour antigens, and also affect the balance between Th-1 and Th-2 phenotypes.^50,51^

Multivariable analysis of clinical factors and genetic findings combined identified a lack of visceral metastases, NLR <5 and high SNV count as highly predictive of clinical benefit to ICI with a c-statistic of 0.90 (Table 3, Fig. 3). Interestingly, a dose–response relationship was also identified between the number of criteria associated with benefit and observed response: (1) 0 of 12 patients (0%) meeting none of the above criteria achieved clinical benefit, (2) 4 of 20 patients (20%) meeting either one of the three criteria had CB, (3) 10 of 13 (77%) patients satisfying two of the three criteria achieved CB and (4) 10 of 10 patients (100%) who met all three criteria developed CB with five PRs and three CRs. Clearly two of these variables are assessed in routine clinical care, and tumour mutation analysis is increasingly commonplace for oncologic care. Notably, these three variables were not associated with clinical benefit in patients that received a taxane. Hence, these results suggest that this three-factor model is specifically predictive of benefit to ICI therapy in mUC.

Aneuploidy is a common tumour feature and has been associated with decreased response rates to ICIs in several tumour types.^41^ Here we used CNV count, including both homozygous deletions of tumour suppressors and high amplification of proto-oncogenes, as a quantitative measure of aneuploidy. In our analysis, both CNV count and CDKN2B homozygous deletions strongly associated with lack of response to ICI therapy. To our knowledge, this correlation with *CDKN2B* deletions has not been reported in prior studies and may be intrinsic to mUC or be of broader relevance to other cancer types. *CDKN2A* and *CDKN2B* are located on chromosome 9p, and commonly deleted in bladder cancer, as well as other cancer types. 9p deletions may extend to multiple other genes on 9p, including IFN and related pathway genes, so that this association may reflect perturbation in IFN signalling that contributes to immune exhaustion and lack of benefit from ICI therapy.

This retrospective analysis has several limitations. First, the relatively small number of patients studied limits the statistical power of our analysis. Second, we included patients who received ICIs in the first-line as well as second-line setting; however, we assessed whether this difference contributed to differences in response, and it was not statistically significant. Third, patients were treated with different ICI regimens that may have variable efficacy. Fourth, most of the tumours subject to mutation analysis were primary lesions, and this eliminates the ability to detect clinically actionable alterations found exclusively in metastatic specimens. Nonetheless, this reflects common clinical practice. Fifth, Oncopanel does not cover all genes known to be mutated at significant frequency in UC,^37^ including several genes that are commonly subject to amplification (*E2F3, SOX4* and *PPARG*). Sixth, we were unable to determine with certainty whether variants were somatic versus germline and could not prove the functional effect of the missense variants identified. Finally, we did not have tumours from patients to perform PD-L1 immunohistochemistry (IHC); however, given that IHC for PD-L1 has not been consistent as a prognostic factor for response to PD-1/PD-L1 inhibitors,^52,53^ we chose to focus on tumour genomics as a more robust and reproducible analysis that can have many clinical implications for management of mUC. High TMB has been reported to be associated with response to atezolizumab post platinum.^54^ Nonetheless, our findings are hypothesis-generating, and should be tested in larger and more uniformly treated cohorts.

## Conclusion

In this cohort of 62 mUC patients treated with ICI therapy, both a high and a low CNV count are strongly associated with response. Multivariable analysis identified lack of visceral metastasis, low NLR and high SNV as being independently predictive of clinical benefit to ICI but not to taxane-based chemotherapy. We used these features to generate a prediction score, which was highly correlated with clinical benefit [AUC (95% CI) = 0.90 (0.80, 0.99)]. These results highlight the power of combining readily available clinical and laboratory data with panel DNA sequencing data to stratify response to ICI in mUC. External validation of this predictive model in other cohorts is warranted.

## Supplementary information

Supplementary Tables

Supplementary Figures

## Data Availability

The datasets supporting the conclusions of this article are included within the article and its Supplementary material.
