# Supplementary material for: A model combining clinical and genomic factors to predict response to PD-1/PD-L1 blockade in advanced urothelial carcinoma
Source: Br J Cancer. 2019 Dec 20;122(4):555–63. doi: 10.1038/s41416-019-0686-0 (PMC7028947; doi:10.1038/s41416-019-0686-0)
Supplement: Supplementary file 2 — Supplementary Figures [file 41416_2019_686_MOESM2_ESM.pdf]

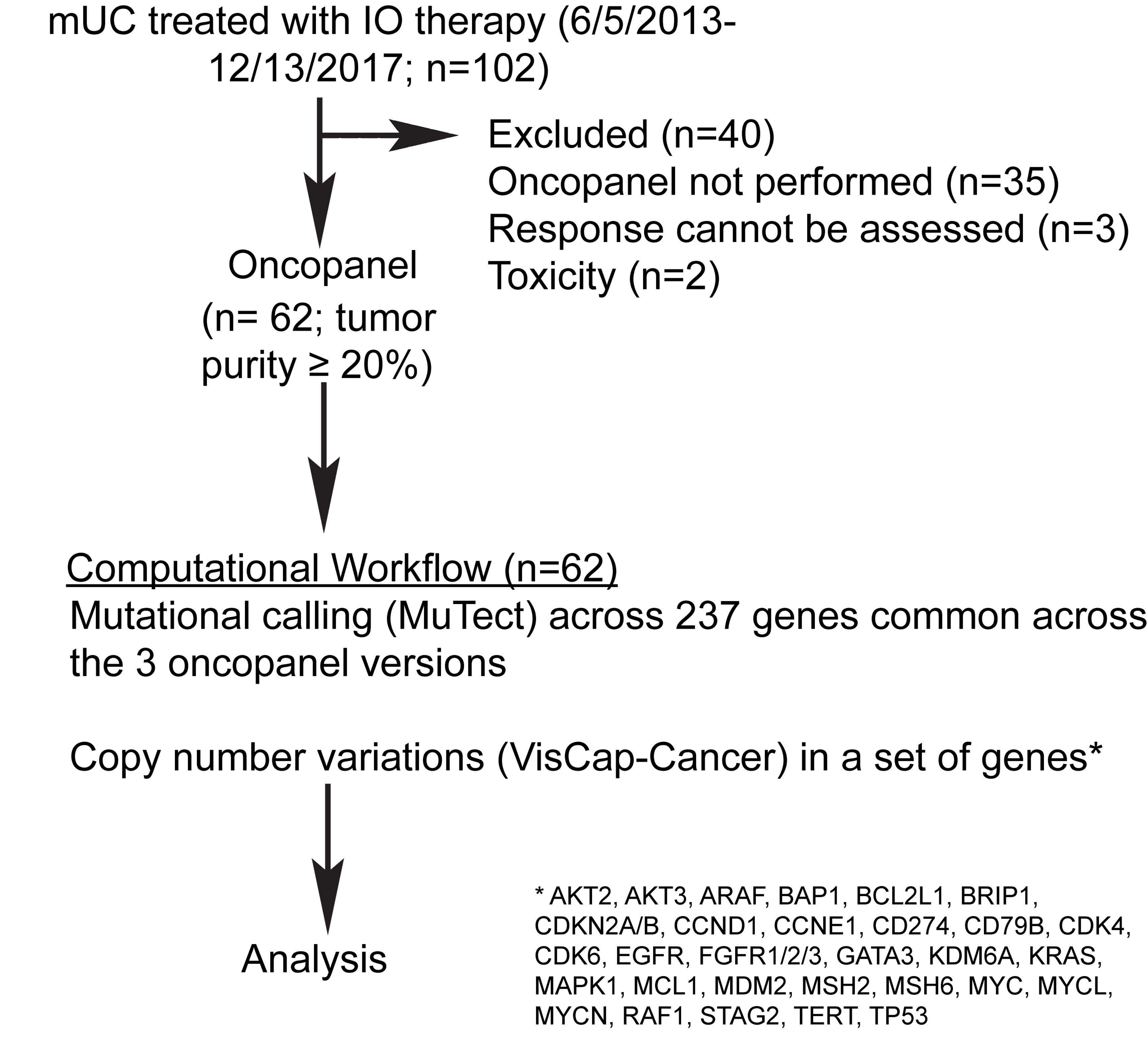

Supplementary Figure 1: Flow diagram of the study.

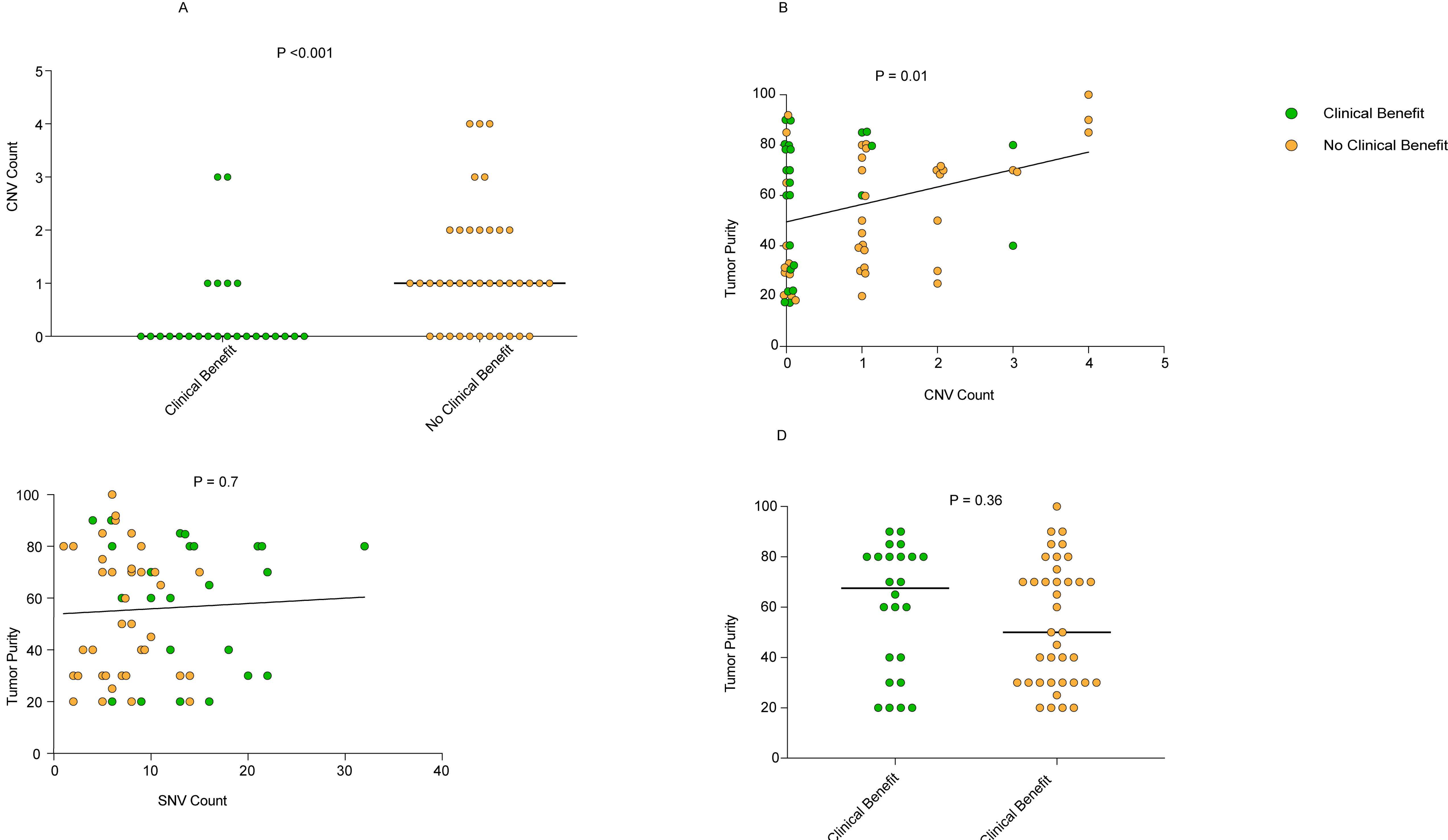

Supplementary Figure 2: Relationships among tumor purity, SNV and CNV counts, and clinical benefit.
(A) Dot plot of CNV count according to CB vs. NCB status;
(B) Correlation between tumor purity and CNV count;
(C) Correlation between tumor purity and SNV count;
(D) Dot plot of tumor purity according to CB vs. NCB. Each dot in each panel represents a single patient.

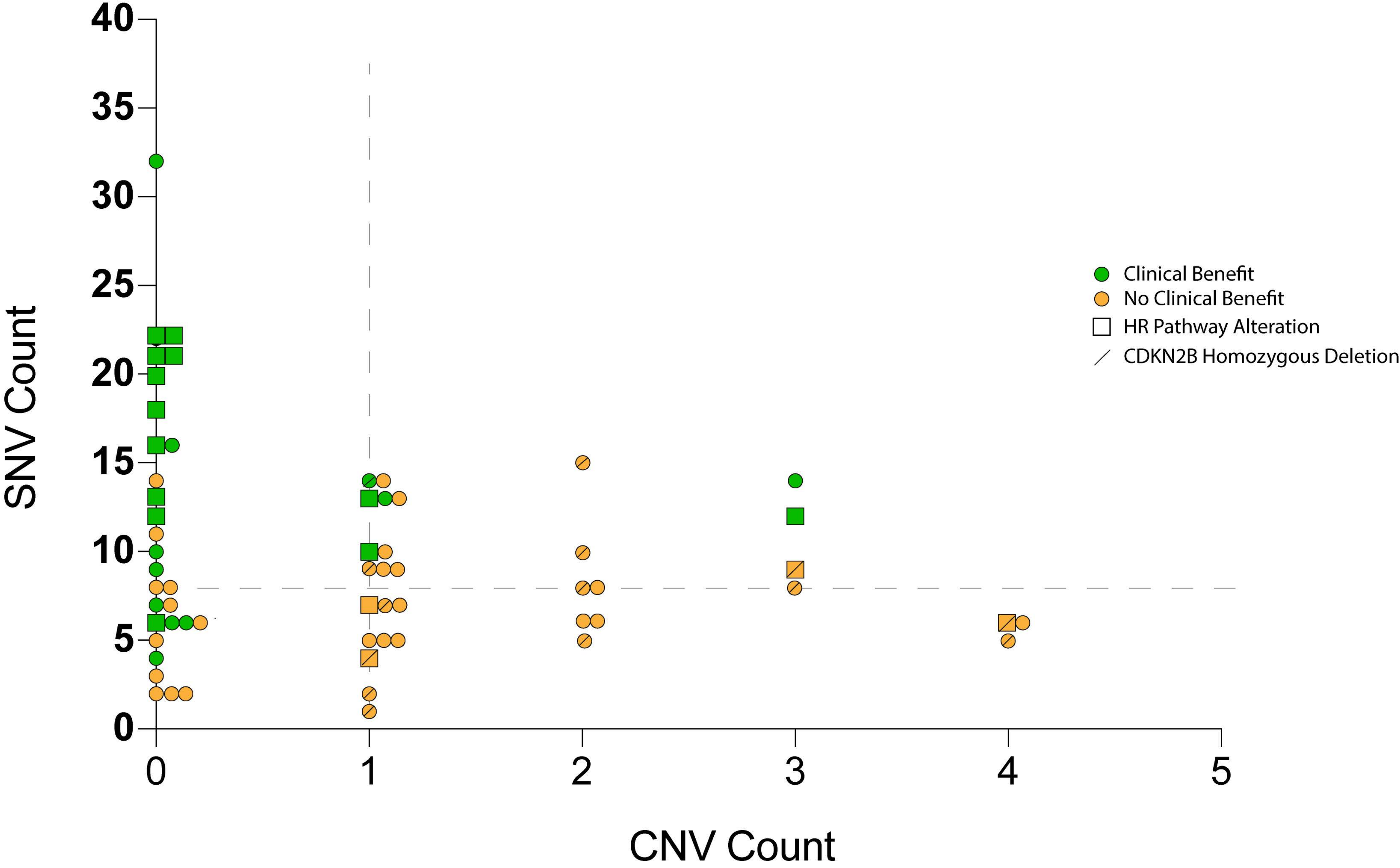

Supplementary Figure 3: Plot of SNV count, CNV count, and clinical benefit. Dotted horizontal and vertical lines denote the median SNV and CNV counts, respectively. Each dot represents a single patient. Clinical benefit, no clinical benefit, presence of an HR pathway alteration (using the more inclusive method for mutation calling), and CDKN2B homozygous deletion are all shown.

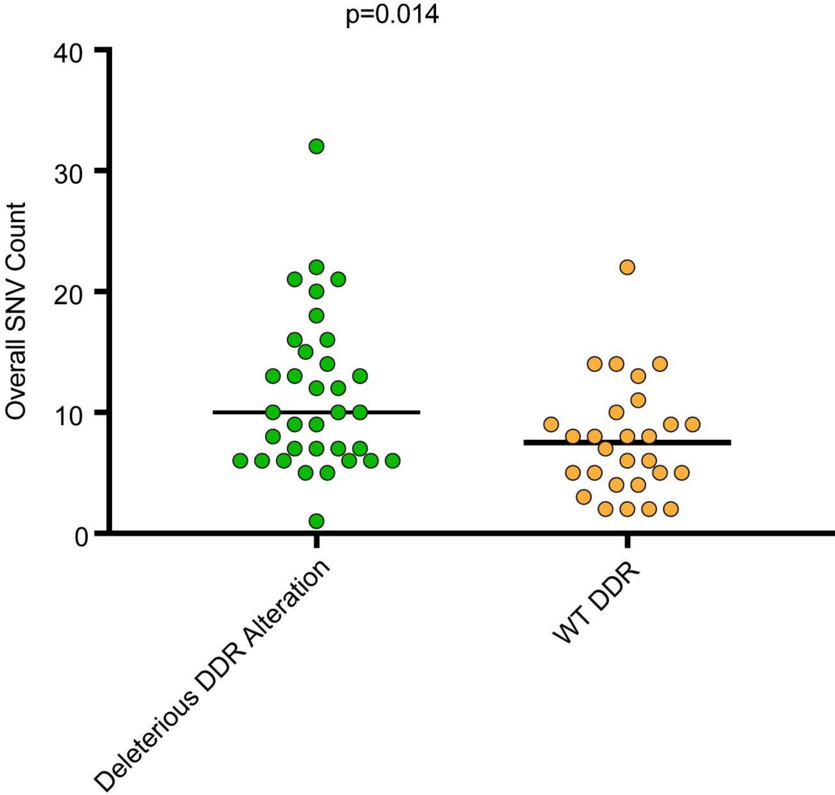

Supplementary Figure 4: Plot of overall SNV count according to presence or absence of a deleterious DDR alteration, using the more inclusive mutation calling method. Each dot represents a single patient.

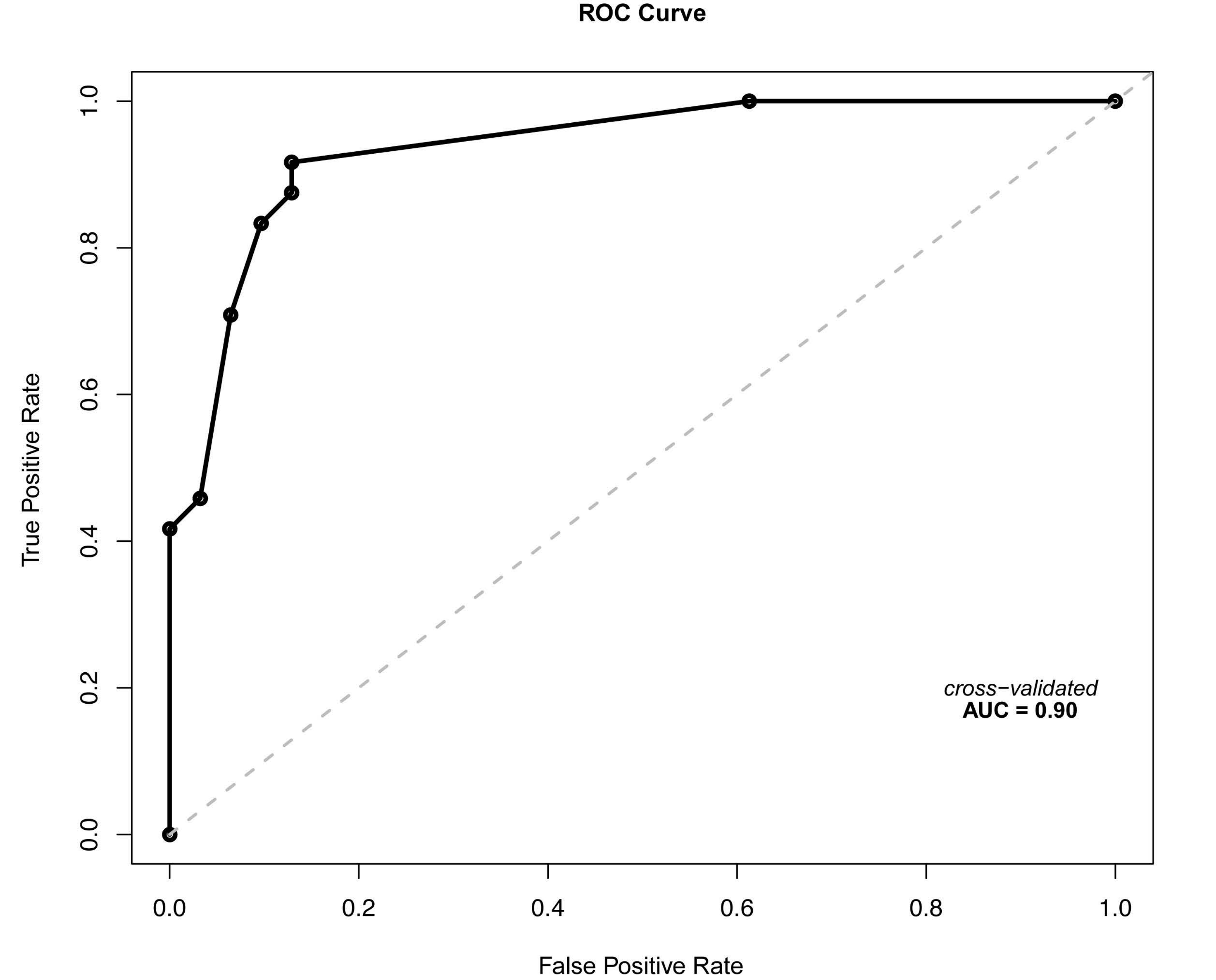

Supplementary Figure 5: Receiver operating characteristics (ROC) curve to assess the sensitivity and specificity of the model combining NLR (<5), lack of visceral metastasis, and SNV count (≥10), for clinical benefit.

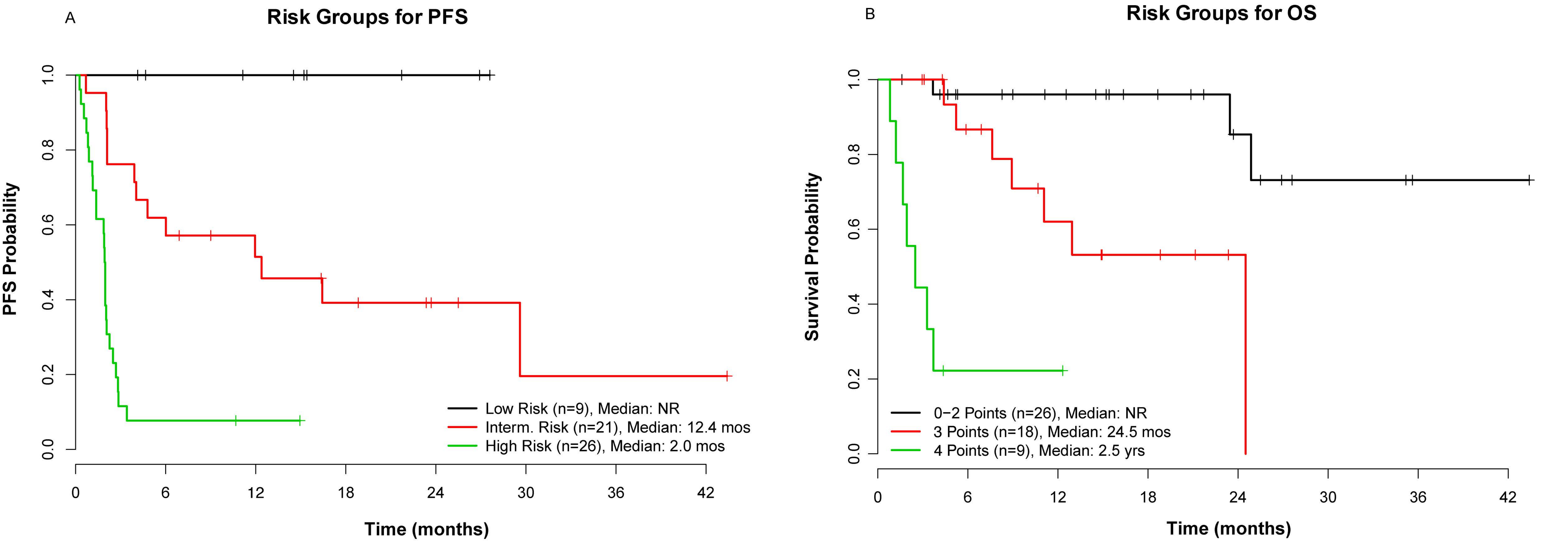

Supplementary Figure 6: Survival curves for UC patients treated with ICI, using combined scores of clinical and genetic features. (A) PFS for three risk groups: Low risk < -0.29; -0.29 ≤ intermediate risk <1.54; high risk ≥ 1.54. A prognostic index was calculated for each patient that is the sum of the product of the patient's risk factor combination and betas (Table S5.1). (B) OS for three risk groups: Low, weighted composite risk score of 0-2; intermediate, weighted composite risk score of 3; high, weighted composite risk score of 4. Risk score components for OS included presence of visceral metastasis (point score of 2), neutrophil-to-lymphocyte ratio >5 (point score of 1), and ECOG PS ≥1 (point score of 1) (Table S5.2).

Supplemental Figures

Figure S1. Flow diagram of the study.

Figure S2. Relationships among tumor purity, SNV and CNV counts, and clinical benefit.

(A) Dot plot of CNV count according to CB vs. NCB status;

(B) Correlation between tumor purity and CNV count;

(C) Correlation between tumor purity and SNV count;

(D) Dot plot of tumor purity according to CB vs. NCB. Each dot in each panel represents a single patient.

Figure S3. Plot of SNV count, CNV count, and clinical benefit. Dotted horizontal and vertical lines denote the median SNV and CNV counts, respectively. Each dot represents a single patient. Clinical benefit, no clinical benefit, presence of an HR pathway alteration (using the more inclusive method for mutation calling), and CDKN2B homozygous deletion are all shown.

Figure S4. Plot of overall SNV count according to presence or absence of a deleterious DDR alteration, using the more inclusive mutation calling method. Each dot represents a single patient.

Figure S5. Receiver operating characteristics (ROC) curve to assess the sensitivity and specificity of the model combining NLR (<5), lack of visceral metastasis, and SNV count (≥10), for clinical benefit.

Figure S6. Survival curves for UC patients treated with ICI, using combined scores of clinical and genetic features. (A) PFS for three risk groups: Low risk < -0.29; -0.29 ≤ intermediate risk <1.54; high risk ≥ 1.54. A prognostic index was calculated for each patient that is the sum of the product of the patient's risk factor combination and betas (Table S5.1). (B) OS for three risk groups: Low, weighted composite risk score of 0-2; intermediate, weighted composite risk score of 3; high, weighted composite risk score of 4. Risk score components for OS included presence of visceral metastasis (point score of 2), neutrophil-to-lymphocyte ratio ≥5 (point score of 1), and ECOG PS ≥1 (point score of 1) (Table S5.2).

Supplementary Material 1. Detailed description of the adaptive least absolute shrinkage and selection operator (ALASSO) approach, internal validation methodology, and prognostic index.

Supplementary Material 1. Detailed description of the adaptive least absolute shrinkage and selection operator (ALASSO) approach, internal validation methodology, and prognostic index.

Statistical Analysis

The adaptive least absolute shrinkage and selection operator (ALASSO) approach:

Due to the limited sample size relative to the number of clinical and genomic features, a shrinkage regression approach, ALASSO[1], was used for variable selection. ALASSO is able to shrink coefficient estimates to 0 thereby selecting those variables with meaningful association with outcomes. The optimal tuning parameter for the ALASSO method was determined, from a range of values, through a 10-fold cross-validation. The tuning parameter is the value that maximizes appropriate model goodness-of-fit measures (deviance and partial-likelihood for logistic and Cox regression respectively). Adaptive weights used in the ALASSO method were coefficients obtained from fitting univariable and multivariable models. Regression coefficients were estimated in univariable analysis, separately for clinical and genomic variables, using binary logistic (for NCB vs CB) and Cox proportional hazards (for PFS & OS) regression models, for each variable selected by the ALASSO method. Using a 2-sided $p \leq 0.05$ criterion, variables from univariable analyses were selected for inclusion in the multivariable model with a stay criterion of ≤ 0.10; the same stay criterion was used for the combined model containing clinical and genomic variables. Model discrimination performance was assessed using the area under the ROC curve (AUC), referred to as c-statistic (or c-index) for PFS & OS. Regression model assumptions were checked.

Internal validation of the model:

For internal validation of model discrimination performance, 20 replications of outcome-stratified 5-fold cross-validation estimate of area under the ROC curve (AUC) was reported for the final logistic regression model; 95% confidence interval for this estimate was also obtained[2] while bias corrected estimates of c-index from 200 bootstrap samples were reported for PFS and OS[3].

Prognostic index:

Prognostic index= (1.63 x αvisceral metastasis [Yes, α=1; No, α=0]) + (0.28 x βplatelet count/100 [β=continuous number]) + (-0.11 x ωSNV count [ω =continuous number]) + (0.28 x δCNV count [δ=continuous number]).

References

1. Zou H. The Adaptive Lasso and Its Oracle Properties. *Journal of the American Statistical Association* 2006; **101**: 1418-29.
2. LeDell E, Petersen M, van der Laan M. Computationally efficient confidence intervals for cross-validated area under the ROC curve estimates. *Electron J Stat* 2015; **9**(1): 1583-607.
3. Harrell FE. Regression modeling strategies : with applications to linear models, logistic and ordinal regression, and survival analysis. Springer series in statistics,. Second edition. ed. Cham: Springer,; 2015. p. 1 online resource (xxv, 582 pages).
